# Salivary expression of lncRNA DQ786243 and IL-17 in oral lichen planus: case–control study

**DOI:** 10.1186/s12903-022-02277-0

**Published:** 2022-06-18

**Authors:** Engy Abdeldayem, Laila Rashed, Shereen Ali

**Affiliations:** 1 Department of Oral Medicine and Periodontology, Faculty of Dentistry, Cairo University, 11 El-Saraya Street, Manial, Cairo, 11553 Egypt; 2 Department of Medical Biochemistry and Molecular Biology, Faculty of Medicine, Cairo University, Cairo, Egypt

**Keywords:** lncRNA DQ786243, IL-17, Oral lichen planus, Saliva

## Abstract

**Background:**

A growing number of studies has investigated IL-17 in OLP. However, its exact role and interactions are not fully determined. In addition, the literature investigating its salivary expression is limited. The scarcity in the literature studying lncRNAs was noticed, particularly with regards to correlating them with cytokines in OLP. In the current study, the salivary expression of lncRNA DQ786243 and IL-17 was assessed among different forms of OLP.

**Methods:**

The study included 52 participants in four equal groups: reticular OLP, erythematous OLP, ulcerative OLP, and control group. All eligible OLP patients underwent conventional oral examination, along with basic charting of their demographic data, pain intensity using a visual analogue scale, and clinical evaluation using the Thongprasom et al. scale. The salivary expression of lncRNA DQ786243 and IL-17 was evaluated for all participants using qRT-PCR. Unstimulated whole saliva samples were used. Data were analyzed for statistical significance.

**Results:**

No statistically significant difference was observed when comparing the mean age and gender distribution of the studied groups. A statistically significant difference was detected when comparing pain and clinical scores in the three OLP forms. The highest expression of both salivary biomarkers was noticed in ulcerative OLP, followed by erythematous OLP and reticular OLP, then the controls, with a significant difference between the studied groups. Upon comparing the salivary expression of DQ786243 in ulcerative and erythematous OLP, no significant difference was detected. No significant difference was detected when comparing salivary expression of IL-17 in erythematous OLP to the other OLP forms.

**Conclusions:**

The salivary expression of lncRNA DQ786243 and IL-17 was upregulated in OLP compared to healthy individuals. Besides, their expression increased when the severity of OLP was at its highest level in ulcerative OLP. There was a positive correlation between DQ786243 and IL-17.

*Trial registration* The protocol was registered at ClinicalTrials.gov (NCT04503824). The date of registration is 07/08/2020.

## Introduction

Oral lichen planus (OLP) is one of the most common oral mucosal diseases. It is presented in different clinical forms, basically as reticular white lesions, red erythematous lesions, or ulcerative areas [1, 2]. Several factors are implicated in the etiology, and several hypotheses are proposed for its pathogenesis. Although the exact etiopathogenesis of OLP is unknown, there is consensus on the fundamental role of the immune dysregulation in the pathogenesis of OLP. Cytokines are still gaining attention in various research [3–5]. Moreover, it is confirmed that the immunological abnormalities influencing the pathogenesis and progression of OLP are caused by an imbalance of Th1/Th2 cytokines. More T cell subsets are studied for their role in OLP, including TH17 cell and its signature cytokine, interleukin (IL)-17 [1, 2]. TH17 cells and IL-17 were first considered part of the innate immunity, mainly the recruitment of neutrophils and macrophages to clear extracellular pathogens. Then, their role in inflammatory and auto-immune disorders was revealed. IL-17 promotes the secretion of potent pro-inflammatory cytokines, such as IL-1, IL-6, IL-22, IL-26, and TNF-α [2, 4]. Studies investigating serum and tissue expression of IL-17 in OLP are conducted [6–14], whereas the literature investigating salivary expression is limited [15]. The exact role and interactions of IL-17 in OLP are still undetermined. Subsequently, more studies are encouraged to further investigate this cytokine in OLP [2, 4].

Although their properties are less well understood, research showed that long non-coding RNAs (lncRNAs) play essential functions in cell biology, including stem cell development, cell differentiation, cell proliferation, and death [16, 17]. One of the recently introduced lncRNAs is DQ786243, which is verified in some malignancies, Crohn's disease, and other autoimmune diseases [18–20]. Studies noticed its upregulation in gastric, colorectal, and ovarian tumors. Studies also observed that silencing DQ786243 inhibits cell proliferation, colony formation, migration, and invasion, by promoting cell apoptosis and arresting cells in the G1/G0 phase, thus acting as a tumor promoter in ovarian cancer cell lines [21–23]. A recent study investigated DQ786243 in OLP, and assessed its expression and effects on the modulation of Treg cells. DQ786243 was assumed to mediate ILs, including IL-17 [24].

Saliva-omics techniques appear to be a promising subject of research in the era of precision medicine. Hence, researchers aim to detect salivary biomarkers, and apply them in clinical practice. Saliva contains locally and systemically produced metabolites and proteins that can be used as diagnostic biomarkers for various disorders. Saliva is also advantageous to the blood since it reduces the risk of injury [25, 26]. Therefore, the present paper assessed the salivary expression of lncRNA DQ786243 and IL-17 among different forms of OLP.

## Methods

The current case–control study is composed of four groups: reticular OLP, erythematous OLP, ulcerative OLP, and healthy controls. Each group included thirteen participants, who were recruited from the Diagnostic Center, as well as the Clinics of the Oral Medicine and Periodontology Department, at the Faculty of Dentistry, Cairo University, during the period from August 2020 to August 2021. The study was conducted following the principles of the Helsinki Declaration, and was approved by the Research Ethics Committee of the Faculty of Dentistry, Cairo University (Code: 20215). The protocol was registered at ClinicalTrials.gov (NCT04503824).

The clinical and histopathological diagnosis of OLP was conducted according to the modified WHO criteria [27]. Patients who agreed to participate in the study, and accepted to sign the informed consent were included. On the other hand, patients suffering from any systemic disease, local inflammatory disease, or infection were excluded. In addition, pregnant and lactating women, as well as smokers were excluded to avoid any potential confounders or effect modifiers that could affect the expression of the studied biomarkers. Participants of the control group were selected to match OLP patients in age and gender to avoid their confounding effect. Eligible patients were enrolled in a consecutive order to avoid the risk of selection bias.

All eligible OLP patients underwent conventional oral examination, along with basic charting of their demographic data, pain intensity, and clinical score. Pain intensity was evaluated using the visual analogue scale (VAS). VAS ranged from 0 to 10, with 0 indicating "no pain," and 10 indicating "pain as bad as you can imagine" [28]. The clinical evaluation of lesions was scored using the Thongprasom et al. scale. In the Thongprasom et al. scale, lesions were scored as follows: 0 indicated no lesions and normal mucosa; 1 indicated mild white striae with no erythematous area; 2 indicated white striae with atrophic area < 1 cm^2^; 3 indicated white striae with atrophic area > 1 cm^2^; 4 indicated white striae with erosive area < 1 cm^2^; and 5 indicated white striae with erosive area > 1 cm^2^ [29].

The salivary expression of lncRNA DQ786243 and IL-17 was evaluated for all participants using qRT-PCR. For saliva sampling, unstimulated whole saliva was used with a passive drooling technique, because it is less sensitive to flow rate and pH than stimulated saliva. In addition, it contains fewer bacteria, and it is less prone to inconsistent concentrations of salivary biomarkers compared to stimulated saliva [30]. Participants were asked to refrain from eating, drinking, or using saliva stimulators for one hour before sample collection. Participants were instructed to swallow, then tilt their head forward, and expectorate saliva into a centrifuge tube for five minutes without swallowing. Saliva samples were frozen at -70 °C, until analysis. Saliva samples were centrifuged at 4500 gm for fifteen minutes.

Total RNA was isolated using a Qiagen kit (Qiagen, USA) according to the instructions of the manufacturer. The total RNA (0.5–2 μg) was used for cDNA conversion, using high-capacity cDNA reverse transcription kit (Fermentas, USA).

Real-time qPCR amplification and analysis were performed using an Applied Biosystem with software version 3.1 (StepOne™, USA). The qPCR assay with primer sets was optimized at the annealing temperature. The primer sequence was F: TAGGCGGACATTGTGGTGAGT, R: CTTCTGCTGGGCTGTTGAGTG for lncRNA DQ786243 and F: ATCCCTCAAAGCTCAGCGTGTC, R: GGGTCTTCATTGCGGTGGAGAG for IL-17. Data were calculated by the 2 − ΔΔCt method.

The sample size was calculated based on a study by Wang et al. [15], and according to the mean difference and SD of both the OLP group and the normal control group. The sample size was calculated with an effect size of 1.177, and a level of significance of 0.05, using a power of 80%. The sample size of 10 subjects per group was increased by 30% to 13 subjects in each group of OLP total of 39 subjects and 13 subjects in the control group. The sample size calculation was achieved using G* Power version 3.1.9.2.

The mean and standard deviation values were calculated for each group, in each test. Data were explored for normality using Kolmogorov–Smirnov and Shapiro–Wilk tests. Age, lncRNA, and IL-17 data showed parametric (normal) distribution, while gender, VAS, and clinical evaluation data showed non-parametric (not-normal) distribution. For parametric data, One-way ANOVA followed by Tukey posthoc test was used to compare more than two groups in non-related samples. An independent sample t-test was used to compare two groups in non-related samples. Pearson correlation test was used to detect the correlation between different parameters. For non-parametric data, the Kruskal Wallis test was used to compare between more than two groups in non-related samples. Mann Whitney test was used to compare between two groups in non-related samples. Spearman correlation test was used to detect the correlation between different parameters [31–33].

The significance level was set at *P* ≤ 0.05. Statistical analysis was performed with IBM® SPSS® Statistics Version 20 for Windows.

## Results

During the recruitment phase, 80 patients were assessed for eligibility. Eight patients did not fulfill the diagnostic criteria of OLP, five patients suffered from chronic systemic diseases, seven patients did not agree to sign the informed consent, three patients suffered from periodontal diseases, and five patients were smokers. Fifty-two patients were eligible for inclusion. The age of the participants ranged as follows: the age range was 32–55 in the reticular OLP group, 23–66 in the erythematous OLP group, 33–58 in the ulcerative OLP group, and 32–56 in the controls. Regarding gender distribution, 8 females and 5 males participated in the OLP groups, while 7 females and 6 males participated in the control group. No statistically significant difference was detected when comparing the mean age and gender distribution of the studied groups (Table 1).

**Table 1:** Characteristics and measured variables

Variables	Control group	Reticular OLP	Erythematous OLP	Ulcerative OLP	*P* value
Age	42.92 ± 7.54	48.69 ± 6.09	43.23 ± 13.24	48.85 ± 6.99	0.166
Gender (M:F)#	6:7 (46.2–53.8%)	5:8 (38.5–61.5%)	5:8 (38.5–61.5%)	5:8 (38.5–61.5%)	0.972
Pain—VAS	–	0.308 ± 0.630^c^	5.385 ± 1.502^b^	8.538 ± 1.450^a^	< 0.001*
Clinical score—Thongprasom et al	–	1.00 ± 0.00^c^	2.385 ± 0.506^b^	4.692 ± 0.480^a^	< 0.001*
Salivary lncRNA DQ786243	1.02 ± 0.02^c^	3.67 ± 1.30^b^	5.41 ± 2.03^a^	6.34 ± 2.02^a^	< 0.001*
Salivary IL-17	1.01 ± 0.01^c^	6.86 ± 1.95^b^	8.28 ± 2.57^ab^	10.08 ± 3.19^a^	< 0.001*

*M* male, *F* female, *VAS* visual analogue scale, *lncRNA* long non-coding RNA, *IL* interleukin

#Count and percentage; *significant (*P* ≤ 0.05); Means with different letters in the same row indicates significant difference

Concerning the two studied salivary biomarkers, the highest expression was noticed in ulcerative OLP, followed by erythematous OLP, then reticular OLP, while the lowest expression was found in the controls (Table 1). For lncRNA DQ786243, statistical analysis revealed a significant difference between the four studied groups. Upon comparing every two groups, the three forms of OLP demonstrated statistically significant differences when compared to the control group. Reticular OLP showed a statistically significant difference when compared to ulcerative or erythematous OLP. Nevertheless, no significant difference was detected when comparing ulcerative and erythematous OLP. Regarding IL-17, statistical analysis revealed a significant difference between the four studied groups. Upon comparing every two groups, the three forms of OLP demonstrated statistically significant differences when compared to the control group. Reticular OLP showed a statistically significant difference when compared to ulcerative OLP. However, no significant difference was detected when comparing erythematous OLP to reticular or ulcerative OLP (Table 1).

A positive correlation was spotted between all the measured variables, either the scores of the clinical outcome measures or the expression level of the salivary biomarkers (Table 2). In addition, the effect of age and gender on the measured variables was investigated. Gender did not affect the scores of the two clinical outcome measures, although slightly higher mean values were obtained among females. A statistically significant difference in the scores of the two clinical outcome measures was spotted among the age groups, with higher mean values among participants above 40. There was no significant difference in the expression level of the two studied biomarkers in males and females, although marginally higher mean values were obtained among males. Similarly, age did not affect the expression level of the two studied biomarkers, although slightly higher mean values were obtained among participants above 40 (Table 3).

**Table 2:** Correlations between measured variables

Variables	Correlation coefficient	Sig. (2-tailed)
VAS & Thongprasom et al	0.943	< 0.001*
lncRNA DQ786243 & IL-17	0.972	< 0.001*
lncRNA DQ786243 & VAS	0.464	0.003*
lncRNA DQ786243 & Thongprasom et al	0.508	0.001*
IL-17 & VAS	0.399	0.012*
IL-17 & Thongprasom et al	0.443	0.005*

*VAS* visual analogue scale, *lncRNA* long non-coding RNA, *IL* interleukin

*Significant (*P* ≤ 0.05)

**Table 3:** Effect of age and gender on the measured variables

	Gender			Age		
	Females	Males	*P* value	≤ 40 years old	> 40 years old	*P* value
Pain—VAS	4.792 ± 3.501^a^	4.667 ± 3.994^a^	0.895	3.05 ± 2.44^b^	6.71 ± 2.92^a^	< 0.001*
Clinical score—Thongprasom et al	2.750 ± 1.567^a^	2.600 ± 1.682^a^	0.789	1.72 ± 1.18^b^	3.52 ± 1.44^a^	< 0.001*
Salivary lncRNA DQ786243	4.086 ± 2.398^a^	4.144 ± 2.827^a^	0.808	4.88 ± 2.11^a^	5.36 ± 2.11^a^	0.486
Salivary IL-17	6.557 ± 3.859^a^	6.559 ± 4.478^a^	0.970	8.26 ± 2.72^a^	8.53 ± 3.07^a^	0.777

*VAS* visual analogue scale, *lncRNA* long non-coding RNA, *IL* interleukin

*Significant (*P* ≤ 0.05); Means with different letters in the same row indicates significant difference

## Discussion

The rapidly growing research recognizes IL-17 as a central player in the immune system. However, the exact role of IL-17 in OLP is not completely clear, and still debated. More studies are required to fully understand the role of IL-17 in OLP pathogenesis [2, 4]. All studies reported higher serum expression of IL-17 among OLP patients compared to healthy individuals [6–8]. Some studies noticed upregulation in tissue expression of IL-17 among OLP patients [9–12], whereas other studies did not detect this upregulation [13, 14]. Concerning of IL-17 level among different forms of OLP, results were controversial. Some researchers observed higher expression in ulcerative or erythematous OLP compared to reticular OLP in serum [6, 7] and tissues [11]. Others observed no differences in IL-17 expression among OLP forms in serum [8] or tissues [9]. There is a scarcity in the literature exploring the salivary expression of IL-17, with only one research group studying IL-17 salivary levels in OLP [15, 34, 35], to the best of the authors’ knowledge. The mentioned research group detailed the salivary expression of IL-17 in one of their articles [15], and focused on the salivary microbiome in the other two articles [34, 35].

In the current study, all OLP groups had higher levels of IL-17 expression than healthy controls, with a statistically significant difference, when comparing the four groups. Ulcerative OLP had the highest values, followed by erythematous OLP, then reticular OLP, while the lowest values were related to the healthy controls. The three forms of OLP demonstrated a statistically significant difference when compared to the control group. A significant difference was detected between reticular OLP and ulcerative OLP, but no significant difference was noticed when comparing erythematous OLP with either reticular or ulcerative OLP. Wang et al. [15] studied only two forms: ulcerative OLP and reticular OLP. They detected significantly higher salivary concentration of IL-17 in ulcerative OLP compared to reticular OLP and healthy controls, in accordance with the results of the current study. Yet, they did not detect a significant difference between reticular OLP and the controls, which is in disagreement with the results of the present study.

The results of the current study are consistent with most of the literature in light of the agreed upregulation of IL-17 in OLP compared to healthy controls either in tissue, serum, or saliva. The highest expression is detected in ulcerative OLP. Accordingly, it seems that IL-17 could be responsible for maintaining the local inflammatory microenvironment of OLP [4]. Yet, a couple of studies did not detect this upregulation in tissues [13, 14], and a few other did not find a difference in expression among OLP forms in serum [8] or tissues [9]. The differences in the results could be linked to confounding effects or different methodologies among these studies. Confounders, such as age and gender, are the cause for the inconclusive data on the cytokine's activity in OLP [4]. The current research attempted to eliminate any confounding effects by matching the recruited patients. Moreover, the effect of age and gender on the expression of the biomarkers was tested. There was no significant difference between males and females, although marginally higher values were noticed among males. Age had no effect as well, although slightly higher values were obtained for patients above 40.

Cytokines do not act in isolation, rather in sophisticated networks. Therefore, research should focus on the interactions between cytokines and their networks, along with the way they synergize or antagonize one another to impact OLP pathogenesis [14, 26]. A recent systematic review recommended lncRNAs as novel candidates in oral disorders including OLP. Molecular changes were stated to stimulate the production of cytokines, consequently, contributing to the pathogenesis of OLP. However, the scarcity in the literature studying lncRNAs in OLP was noticed. Accordingly, the review encouraged researchers to further investigate lncRNAs in these disorders to unveil their role [36]. At the best of the authors’ knowledge, there is only one study investigating the expression of DQ786243 in OLP [24], concluding that the expression of DQ786243 was significantly upregulated in the CD4+ cells from the peripheral blood of OLP patients compared with controls. Yet, the sample size was limited, with only ten OLP patients. In addition, the differences in expression among different forms of OLP were not mentioned.

In the present study, the salivary expression level of lncRNA DQ786243 showed a statistically significant difference between the four groups, with the highest value recorded in ulcerative OLP, followed by erythematous OLP, then reticular OLP, while the lowest values were in the healthy controls. A significant difference was obtained when comparing reticular OLP with ulcerative and erythematous OLP, whereas no significant difference was detected between ulcerative and erythematous OLP. Thus, DQ786243 was more upregulated in potentially malignant forms of OLP, erythematous, and ulcerative OLP [37]. These results are consistent with the previous studies that considered DQ786243 as an oncogene [21–23].

A positive correlation was revealed between the expression level of IL-17 and pain, as well as clinical scores in the present study. In agreement, Wang et al. [15] stated a similar positive correlation between IL-17 and disease clinical scores. In addition, a positive correlation was found during the current study between the expression level of DQ786243 and pain as well as clinical scores. No previous studies correlated the expression of DQ786243 with pain and clinical scores. Furthermore, the positive correlation between the salivary expression of the two investigated biomarkers and the two clinical parameters led to the observation that DQ786243 and IL-17 might contribute to the course of the disease. In addition, detecting the highest scores—biomarkers and clinical parameters—in ulcerative OLP, followed by erythematous, then reticular OLP supported the link between disease severity and DQ786243 and IL-17. Concerning the correlation between DQ786243 and IL-17, Wang et al. [24] reported that when they overexpressed DQ786243 in the CD4+ cells from normal controls, IL-17 expression was significantly suppressed in these cells. The current study detected a strong positive correlation between the salivary expression levels of DQ786243 and IL-17.

Hence, the present results are more consistent with the well-accepted concept that carcinogenesis and inflammation are linked. The malignant potential of OLP is attributed to the chronic inflammation maintained in the microenvironment of this disease via cytokines and other inflammatory mediators [38, 39]. Further research is still necessary to assess the relationship between the two markers on a larger sample size. The strength of the current study is being the first to investigate the salivary expression level of lncRNA DQ786243 in OLP and among its different clinical forms. The study is the second research to assess the salivary expression of IL-17. The limitations of the present study include the relatively small sample size, and the confounding effect that is inherent in this study type.

## Conclusions

The salivary expression of lncRNA DQ786243 and IL-17 were upregulated in OLP compared to healthy individuals, while their expression increased with the highest level of severity of OLP in ulcerative OLP. There was a positive correlation between DQ786243 and IL-17, and both biomarkers were positively correlated with the pain and clinical scores. The salivary expression of lncRNA DQ786243 and IL-17 was not markedly affected by either age or gender. These results could offer a better understanding of the pathogenesis of OLP. Furthermore, the present study recommends further trials with a larger sample size to decrease the effect of confounders, and reveal more about the diagnostic and prognostic roles of these salivary biomarkers in OLP. Hopefully, the studied biomarkers have revealed diagnostic or therapeutic roles in the future.

## Data Availability

The datasets used and analyzed during the current study are available from the corresponding author on reasonable request.
